# Genotype by environment cultivar evaluation for cassava brown streak disease resistance in Tanzania

**DOI:** 10.1016/j.virusres.2020.198017

**Published:** 2020-09

**Authors:** Rudolph R. Shirima, James P. Legg, Daniel G. Maeda, Silver Tumwegamire, Geoffrey Mkamilo, Kiddo Mtunda, Heneriko Kulembeka, Innocent Ndyetabula, Bernadetha P. Kimata, Dwasi Gambo Matondo, Gloria Ceasar, Edda Mushi, Karoline Sichalwe, Edward Kanju

**Affiliations:** a IITA, Dar es Salaam, Tanzania; b University of Dar es Salaam, Tanzania; c IITA, Kigali, Rwanda; d Tanzania Agricultural Research Institute, Naliendele, Tanzania; e Tanzania Agricultural Research Institute, Ukiriguru, Tanzania; f Tanzania Agricultural Research Institute, Maruku, Tanzania; g IITA, Kampala, Uganda; h Tanzania Agricultural Research Institute, Tumbi, Tanzania; i Tanzania Agricultural Research Institute, Makutupora, Tanzania; j Tanzania Agricultural Research Institute, Kibaha, Tanzania

**Keywords:** Cassava, agro-ecological zone, CBSV, UCBSV, resistance, yield

## Abstract

Cassava brown steak disease (CBSD), caused by Cassava brown streak virus (CBSV) and Ugandan cassava brown streak virus (UCBSV), is the most important biotic constraint to cassava production in East and Central Africa. Concerted efforts are required to prevent further spread into West Africa as well as to reduce losses in areas already affected. The study reported here was part of a five-country (Kenya, Malawi, Mozambique, Tanzania and Uganda) programme that aimed to identify superior cassava cultivars resistant to CBSD and to disseminate them widely in the region. Seventeen tissue-cultured and virus-tested cultivars were evaluated in Tanzania across nine sites with diverse CBSD inoculum conditions. Experiments were planted using an alpha-lattice design and assessments were made of surrounding inoculum pressure, CBSD foliar and root incidence and root yield at harvest. There were large differences in CBSD infection between sites, with greatest spread recorded from the north-western Lake (Victoria) zone. Differences were driven by *Bemisia tabaci* whitefly vector abundance and CBSD inoculum pressure. Both CBSV and UCBSV were almost equally represented in cassava fields surrounding experimental plots, although CBSV predominated in the north-west whilst UCBSV was more frequent in coastal and southern sites. However, the incidence of CBSV was much greater than that of UCBSV in initially virus-free experimental plots, suggesting that CBSV is more virulent. Cultivars could be categorised into three groups based on the degree of CBSD symptom expression in shoots and roots. The seven cultivars (*F10_30R2*, *Eyope*, *Mkumba*, *Mkuranga1*, *Narocass1*, *Nase3* and *Orera*) in the most resistant category each had shoot and root incidences of less than 20%. Fresh root yield differed between sites and cultivars, but there was no genotype by environment interaction for this trait, probably attributable to the large fertility and soil moisture differences between sites. Susceptible cultivars and the local check performed well in the absence of CBSD pressure, highlighting the importance of exploiting quality and yield traits of local landraces in breeding programmes. Overall, our results emphasized the importance of applying a balanced strategy for CBSD management. This should use both improved and local germplasm resources to generate high yielding cultivars for specific end-user traits, and combine the deployment of improved cultivars with phytosanitary control measures including the use of healthy planting material and planting during periods of reduced CBSD infection.

## Highlights

• 17 cultivars from 5 countries evaluated for CBSD resistance at 9 sites in Tanzania.
• Site differences in disease pressure and vector abundance drove CBSD spread.
• Seven cultivars were identified with strong resistance to CBSD.
• CBSD-susceptible cultivars gave high yields at low disease pressure sites.
• CBSD control should combine host plant resistance with phytosanitary measures.

## 1 Introduction

Cassava is an important source of food to many people in the tropics and sub-tropical locations of the world where its key role as a subsistence crop is significant as well as its use in industrial processing (Ceballos et al. 2012). The importance of cassava is further emphasized by the fact that it is perceived as the future food security hope for Africa because it can survive unpredictable climatic conditions that may be exacerbated under future climate change scenarios (Jarvis et al. 2012). Nevertheless, cassava virus diseases continue to cause widespread losses to cassava production throughout East and Central Africa despite large-scale efforts deployed to mitigate their impact. Two of the most important current biotic constraints are the virus diseases: cassava mosaic disease (CMD) caused by cassava mosaic begomoviruses (CMBs) and cassava brown streak disease (CBSD) caused by cassava brown streak ipomoviruses (CBSIs) (Legg et al. 2011, 2015). Although CMD is still prevalent wherever cassava is grown in Africa, its impacts have been largely reduced through planting of resistant cultivars (Manyong et al. 2000). CBSD, however, continues to pose a major threat to Africa’s cassava producers. Only moderate success has been achieved in identifying durable CBSD resistance/tolerance through historical conventional breeding approaches. Important progress has been made using a variety of strategies to engineer resistance/tolerance using transgenic approaches (Yadav et al. 2011; Ogwok et al. 2012; Odipio et al. 2014; Beyene et al., 2016). However, the impact of this work continues to be constrained by the current unfavourable regulatory conditions in most of the countries either directly affected or threatened by CBSD. This situation has forced researchers in the region to continue to rely on conventional breeding approaches (Kaweesi et al. 2014; Kawuki et al. 2016; Tumwegamire et al. 2018), albeit also supported by other biotechnological approaches such as marker-assisted breeding (Amuge et al. 2017; Anjanappa et al. 2018). Two CBSI species: CBSV and UCBSV (Mbanzibwa et al. 2009; Winter et al. 2010) are responsible for the CBSD pandemic and are both widely distributed in the affected areas of East Africa (Mbanzibwa et al. 2009; Winter et al. 2010). Both CMBs and CBSIs are transmitted by the same whitefly vector, *Bemisia tabaci* (Genn.) (Dubern, 1994; Maruthi et al. 2005).

From the time of its first report in the 1930s (Storey, 1936), CBSD remained confined for decades within the coastal lowlands of East Africa and around Lake Malawi (Nichols, 1950). A new outbreak of CBSD, however, spread rapidly from the mid-2000s at locations > 1000 metres above sea level (m.a.s.l) in East Africa (Alicai et al. 2007). This outbreak developed quickly into a pandemic in the Great Lakes region of East and Central Africa. As with the severe CMD pandemic before it, it was considered that the ‘trigger’ for this sudden change in disease epidemiology was the greatly increased abundance of the whitefly vector, *B. tabaci* (Legg et al. 2011, 2014). Later reports highlighted further westwards spread into parts of Central Africa (Bigirimana et al. 2011; Mulimbi et al. 2012; Mulenga et al. 2018), associated primarily with UCBSV. Further CBSD spread to the east has been reported in the Comoros Islands highlighting the spread of both CBSV and UCBSV (Azali et al. 2017). As opposed to the earlier spread of only UCBSV in Central Africa, more recently, mixed infections of CBSV and UCBSV have been reported in north-eastern Democratic Republic of Congo (DRC), albeit at low incidence (Casinga et al. 2019).

It is becoming clear that much of the spread of CBSD is through infected planting material. CBSIs have been shown to be spread by the whitefly vector over relatively short distances, as the semi-persistent mode of transmission means that virus particles are retained by whiteflies for relatively short periods of time (Jeremiah, 2012; Maruthi et al. 2017).

Whereas distribution of quality planting material is vital to the success of cassava production, sustainable seed systems must be implemented in ways that minimize or prevent the propagation of viruses in planting material. These should be applied in such a way that efforts to generate improved germplasm are effectively safeguarded (Dixon et al., 2003; Kawuki et al. 2016). There has been limited progress in developing CBSD-resistant cultivars, and none of the currently available cultivars in East and Central Africa has a high level of resistance to the disease. A recent study on cassava degeneration (Shirima et al. 2019) points out the influence of the environment and planting season as key aspects in the successful evaluation of breeders’ material, highlighting large seasonal differences in whitefly abundance which led to contrasting patterns of disease spread. Several studies have published information on field resistance of cassava cultivars to CBSD using sets of cassava cultivars, but these did not cover multiple locations (Kaweesi et al. 2014; Kawuki et al. 2016; Masinde et al. 2018). There are currently no reports of the response of cassava cultivars to CBSD under contrasting agro-ecological conditions. In order to address this gap in knowledge, the current study therefore evaluated 17 cultivars including one susceptible check from diverse sources at nine sites located in four contrasting acro-ecological zones in Tanzania. Note that in our study, we follow the example of Thresh et al. (1998); Kaweesi et al. (2014) and Kawuki et al. (2016) in using ‘resistance’ to describe a reduced propensity for cassava cultivars to become infected by CBSIs, manifested by a reduced incidence of disease symptoms.

## 2 Material and methods

### 2.1 Cassava cultivars and experimental sites

Sixteen elite cassava cultivars and or clones from Kenya, Malawi, Mozambique, Uganda and Tanzania, hereafter referred to as “cultivars”, and one CBSD-susceptible cultivar (*Albert*) were obtained under the “New Cassava Varieties and Clean Seed to Combat CBSD and CMD Project (5CP) (IITA-Tanzania, 2012; Tumwegamire et al. 2018). Stem cuttings for each cultivar from each country were sent to the UK’s Natural Resources Institute as well as the Kenya Plant Health Inspectorate Services in Nairobi, Kenya for virus indexing and tissue culture (TC) production. Virus-indexed TC plants were mass-multiplied at Genetic Technologies International Limited in Nairobi, Kenya. Following proper plant import/export procedures (Tumwegamire et al. 2018), up to 300 tissue culture (TC) plants per cultivar were hardened off at the Tanzania Agricultural Research Institute (TARI) Kibaha in Coast Region (Pwani) and the TARI station at Maruku in Bukoba (north-western Tanzania). Hardened plants were multiplied in the field at TARI-Makutupora in Dodoma for the TC plants that were hardened at Kibaha, while those hardened at Maruku were multiplied on station. These sites were selected in view of their negligible CBSD inoculum pressure. Multiplication fields were isolated by being situated at distances of more than 300 m from any other cassava field. Plants multiplied at Makutupora were used to plant experimental sites in central, eastern and south- eastern Tanzania while those multiplied at Maruku were used to plant sites in north-western Tanzania.

The timing of the onset of the rainy season in the respective agro-ecological zones where the experiment was conducted predetermined the planting dates. Seven sites were planted between October 2015 and January 2016 while the remaining two sites were planted in April 2016 (Table 1). All sites were maintained under rainfed conditions throughout the growing season. The planting plan followed an alpha-lattice design with two or four plots per block and up to 21 blocks depending on field layout per site. Each plot measuring 6 m by 7 m was planted with 1 m spacing between plants resulting in 42 plants per plot. Blocks were separated by 2 m spaces. The outer lines of each plot were considered as guard rows while the remaining inner lines (20 plants [four lines times five plants]) were considered as the net plot which was used for all field assessments and statistical analyses conducted during the experiment.

**Table 1:** Characteristics of sites used for the evaluation of selected cassava cultivars’ response to cassava brown streak ipomoviruses in Tanzania, 2015-2017

Site	District	Altitude category	*CBSD pressure	Latitude	Longitude	Altitude (m)	Planting date
Bunda	Bunda	Mid	High	−1.9401	33.7802	1271	21-Nov-15
Chambezi	Bagamoyo	Low	High	−6.5554	38.9141	50	09-Apr-16
Chato	Chato	Mid	High	−2.6254	31.7875	1150	07-Dec-15
Hombolo	Dodoma	Mid	Low	−5.9625	35.9823	1038	10-Dec-15
Kizimbani	Zanzibar	Low	Moderate	−6.1060	39.2892	64	11-Apr-16
Maruku	Bukoba	Mid	Low	−1.4180	31.7772	1349	23-Nov-15
Naliendele	Mtwara	Low	Moderate	−10.3848	40.1645	145	23-Dec-15
Suluti	Namtumbo	Mid	Moderate	−10.5436	36.0765	882	15-Dec-15
Ukiriguru	Misungwi	Mid	Moderate	−2.7284	33.0229	1205	19-Nov-15

*Shirima (2019)

### 2.2 Surrounding disease inoculum pressure

At two months after planting (2MAP), cassava fields within a 250 m radius of each of the nine experimental sites were assessed for CBSD incidence and vector abundance. For each surrounding field, the distance between the centre of that field and the central point of the closest edge of the experimental plot was estimated using a GPS unit by walking between these two points. *B. tabaci* adults were counted on the first five fully expanded leaves of the tallest shoot of each of 100 plants selected randomly along two diagonals (50 plants on each) in the field. CBSD incidence was calculated as the proportion of the 100 plants expressing foliar CBSD symptoms. The total number of plants in the field was estimated by counting plants on two adjacent edges of the surrounding field and calculating their product. These data were used to calculate surrounding CBSD index (Surr CBSD index) using the method of Legg et al. (1997). Crop age for each surrounding field was also recorded. Depending on the number of surrounding fields and availability of symptomatic plants, ten asymptomatic and up to fifty CBSD-symptomatic leaf samples were collected per site for detection of CBSIs. The central leaf lobe of the fifth fully open leaf (counting from the shoot tip) was picked and pressed in a wooden herbarium press which was clearly labelled with the field number and site name. Leaf samples were kept dry in this way until required for nucleic acid extraction. During nucleic acid extraction, approximately 35 mg of dried leaf was picked, and total RNA was isolated using an optimized CTAB (cetyltrimethyl ammonium bromide) method with some modifications from the methods of Lodhi et al. (1994) and Maruthi et al. 2002. The resulting RNA was analysed using CBSV- and UCBSV-specific real-time RT-PCR TaqMan assays (Shirima et al. 2017; Adams et al. 2013).

### 2.3 Vector abundance and CBSD symptom assessment in the experimental plots

Vector abundance (*B. tabaci*) was estimated at 2MAP by counting whiteflies on five fully expanded top leaves of the tallest shoot of each of ten plants selected randomly along two alternating plant rows within the net plot. Averages of these counts were calculated as a proxy for the number of insects per plant (whitefly abundance). CBSD shoot symptoms were assessed for all experimental sites at 2MAP and at 12MAP for all sites. CBSD foliar incidence was calculated as the percentage of plants expressing foliar symptoms of CBSD. Data were collected for leaf symptom severity using a scale of 1-5 where 1 = asymptomatic, 2 = mild severity and 5 the most severe symptoms (Gondwe et al. 2003). Severity scores from 2 to 5 were averaged per plot and the resulting value represented the mean severity score for the cultivar planted in that plot. Asymptomatic plants (score 1) were not included in these calculations. Means of the three replications were regarded as “shoot severity” for a given cultivar.

### 2.4 Cassava brown streak ipomovirus testing in leaves and roots

Five CBSD symptomatic plants were randomly tagged along the two alternate rows at 2MAP and used for leaf sample collection for CBSIs testing. Where the number of symptomatic plants was lower than five, or where no symptoms were observable, plants were randomly selected along these alternate rows. Leaf samples once collected were pressed in a wooden herbarium press and preserved dry before further analysis. Fifteen plants were sampled per cultivar (five plants from each replication) making a total of 255 leaf samples collected per site and tested for CBSIs at 2MAP. In total, 135 leaf samples were tested per cultivar across all nine experimental sites.

At 12MAP when the five tagged plants were harvested, root samples were collected whenever symptomatic roots were encountered, following the root cutting procedure described in Section 2.5. On each occasion a ca 500 g sample was chopped from one symptomatic root and another from an asymptomatic root of the same plant. The total number of root samples collected per site depended on the presence of root symptoms. Collected root samples were wrapped in clean aluminium foil and labelled. The labelled samples were placed immediately in a cool box containing ice blocks and temporarily stored in a freezer at −20 °C. When brought to the laboratory (IITA, Dar es Salaam), samples were frozen at −80 °C until further analysis. Additionally, a random sample was collected in a similar way from plots where no root necrosis symptoms were encountered. RNA extraction and virus testing were conducted as described earlier (Section 2.2) whereas for root samples, approximately 200 mg of fresh root sample was used. While testing RNA from leaf samples, pools of five samples per plot were tested and subsequently individual samples were tested from all pools that gave positive results.

### 2.5 Root yield and CBSD root symptoms assessment

At 12MAP cassava plants in the net plot were harvested. Roots from one net plot were pooled together and their composite weight was recorded using a balance. Total root weight per plot was calculated by adding weights of the individual roots from the five tagged plants to the composite weight of the 15 plants. This was converted to tonnes per hectare (t/ha). Root dry matter content (DM) was calculated using the specific gravity method developed by Teye et al. (2011) using roots from three randomly selected plants per plot. Harvest index (HI) was calculated as the ratio of root yield in tonnes per hectare (t/ha) to the total biomass (sum of the total root and shoot yields in t/ha).

CBSD root necrosis symptoms were assessed for the five tagged plants by making five cross-sectional cuts in each of the roots harvested from the five tagged plants. CBSD symptoms were then scored using a scale of 1-5 where 1 = healthy, 2 = mild and 5 = severe corky necrotic symptoms with root constrictions (Hillocks and Thresh, 2000). Additionally, roots from the remaining 15 net plot plants were piled up, cut individually and assessed for CBSD symptoms as described. Data from these two sets were pooled and calculations made to get total root incidence and unusable root incidence (Ndyetabula et al. 2016).

### 2.6 Data analysis

Analysis of variance, linear regression and correlations were performed using the General Linear Model and correlation analysis procedures of the Statistical Analysis System (SAS, Institute Inc. Cary, NC, USA, version 9.4). Means were separated using the Student-Newman-Keuls Test imbedded in the General Linear Model Procedure of SAS. One-way Anova was employed to perform pairwise comparisons of CBSD root severity means between sites and means were separated using the Holm-Sidak procedure at the P < 0.05 level. Correlation analyses were used to examine relationships between CBSD leaf and root incidences, CBSD incidences versus yield parameters as well as the relationship between foliar CBSD incidences recorded in trial plots and the CBSD inoculum pressure in surrounding fields (Surr CBSD index). Surr CBSD index is composed of three variables: plant population, CBSD incidence and the distance of surrounding fields from the trial plot. The effects of Surr CBSD index and whitefly abundance for predicting CBSD foliar incidences in the trial plots were examined using multiple regression analyses.

## 3 Results

### 3.1 Surrounding CBSD inoculum pressure

**Fig. 1:**
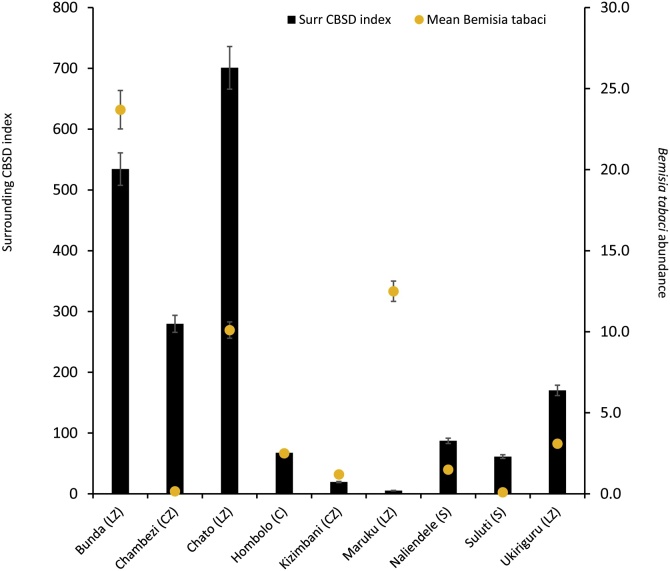
Cassava brown streak disease (CBSD) inoculum pressure at nine experimental sites in Tanzania, 2016. **C** = central zone, **CZ** = coastal zone, **LZ** = lake zone, **S** = southern zone, **Surr CBSD inc** = mean CBSD shoot incidence in surrounding fields, **Surr CBSD index** = an index of the level of CBSD inoculum pressure surrounding the experimental site, **Mean *Bemisia tabaci*** = *Bemisia tabaci* abundance (Insects were counted from five fully expanded shoot tip leaves of the tallest shoot of each of ten plants selected randomly in a net plot. Averages of these counts were used to estimate the number of insects per plant [i.e. abundance] and to calculate the overall means per site.

Contrasting levels of CBSD inoculum pressure (Surr CBSD index) were observed amongst the surrounds of the sites used in this study. Although the Lake Zone (LZ) had the two sites with the highest Surr CBSD index values (Chato, 700.9 and Bunda, 534.3; Fig. 1), the high degree of variability of Surr CBSD index within sites in each zone was such that there was no overall significant difference between the two zones. The situation was similar for whitefly abundance where although all LZ sites had higher whitefly abundances than all Coastal Zone (CZ) sites, the high degree of variability meant that there was no statistically significant difference between the two groups of sites.

There was no significant correlation between either distance (*P =* 0.58) or plant population (*P =* 0.57) of surrounding fields with foliar incidence of CBSD in trial plots. There were, however, significant correlations of foliar incidence of CBSD in trial plots with whitefly abundance (*P =* 0.050) and CBSD incidence in surrounding fields (*P =* 0.003). However, the factor giving the most strongly significant correlation with CBSD incidence in trial plots was the surrounding CBSD index (which combines plant population and distance with CBSD incidence in surrounding fields) (*P =* 0.0005). This is a clear confirmation of the value of the surrounding CBSD index for predicting subsequent CBSD spread into initially CBSD-free trial plots. Additionally, multiple regression analyses demonstrated the value of combining both surrounding CBSD index and whitefly abundance (in surrounding fields) for predicting subsequent foliar CBSD incidence in trial plots (r2 = 0.94, F = 45.7, *P =* < 0.001): the expression generated was: CBSD foliar incidence = -2.755 + (0.0291 * Surr CBSD index) + (0.189 * *B. tabaci* abundance).

Overall, CBSI infections within surrounding fields were detected in relatively equal proportions: 55% CBSV and 49% UCBSV. The pattern was similar at the high inoculum pressure LZ sites of Bunda and Chato, as well as at Naliendele in the southern zone (S) whereas varying proportions of the two viruses were observed at the other sites (Table 2). Single infections with CBSV were higher than those with UCBSV at Kizimbani and Ukiriguru, whilst for UCBSV, single infection frequencies were higher than those of CBSV at Chambezi, Hombolo and Suluti (Table 2). Overall, the proportion of positive tests for CBSIs was greatest at Bunda (100% total) followed by Chato, both of which had different percentages of the samples infected by CBSV-alone, UCBSV-alone and mixed infections (CBSV and UCBSV). Other sites with relatively high percentages of infected samples were Ukiriguru in LZ, Chambezi in CZ, Suluti in southern Tanzania, and Naliendele in south-eastern zone. The remaining sites had less than 70.0% of infected samples: Kizimbani in CZ, Hombolo in central Tanzania and Maruku in the LZ with only CBSV alone (Table 2). Although the relationship between the level of infection in surrounding fields and experimental plots is clear for the high inoculum pressure sites in Bunda and Chato, it is noteworthy that some of the other sites (e.g. Chambezi and Ukiriguru) with relatively high infection levels in the surrounding fields had low infection levels within the experimental plots. Although both virus species occurred frequently in fields surrounding experimental sites in all regions of Tanzania, there was a generally greater frequency of CBSV in the LZ whilst UCBSV was more prevalent at sites in central, CZ and southern parts of the country.

**Table 2:** Proportions of cassava brown streak ipomoviruses infections in the surroundings of experimental sites in Tanzania

Site	*No. samples	CBSIs	%CBSV	%UCBSV	%CBSV only	%UCBSV only	%Mixed infections
Bunda	61	CU	89	82	18	10	72
Chambezi	60	CU	57	70	13	27	43
Chato	60	CU	77	78	13	13	63
Hombolo	58	CU	10	47	5	41	5
Kizimbani	69	CU	58	13	52	7	6
Maruku	20	C	45	0	45	0	0
Naliendele	54	CU	50	50	24	24	26
Suluti	60	CU	30	65	13	48	17
Ukiriguru	65	CU	80	37	46	8	31
**Overall Mean/Total**	**507**		**55**	**49**	**26**	**20**	**29**

*Number of plants tested at each site. Cassava brown streak ipomoviruses (CBSIs) were detected from leaf samples using real-time RT-PCR TaqMan assays according to the protocols published in Adams et al., 2013 and Shirima et al. 2017. **%CBSV** = percentage of samples infected by CBSV, **%UCBSV** = percentage of samples infected by UCBSV, **%CBSV only** = percentage of samples infected by CBSV alone, **%UCBSV only** = percentage of samples infected by UCBSV alone, **%Mixed infections** = percentage of samples infected by both CBSV and UCBSV.

### 3.2 Vector abundance and CBSD symptoms in the experimental plots

*B. tabaci* abundance varied significantly across sites (F = 113.78, *P* < 0.0001), where the highest whitefly numbers (more than 10 insects per plant) were recorded in decreasing order from Bunda, Chato and Ukiriguru in north-western Tanzania (Table 3). Differences in vector abundance amongst cultivars were significant (F = 1.88, *P* < 0.04). The highest *B. tabaci* abundance was recorded for cultivar *Sagonja* (28.3 insects per plant) with the least for *Mkumba* (9.4) and *Mkuranga1* (10.3) (Table 4).

**Table 3:** *Bemisia tabaci* and cassava brown steak disease symptoms of selected cultivars planted at nine sites in Tanzania, November 2015-November 2016 (north-western Tanzania), December 2015-December 2016 (central and southern Tanzania) and April 2016-April 2017 (coastal Tanzania)

Site	N	Mean *Bemisia tabaci*	CBSD 2MAP	CBSD 12MAP	Root incidence	Unusable root inc.	CBSIs in roots
Bunda	51	79.6a	2.2a	15.9b	31.6a	23.8a	74.7
Chambezi	50	4.3ef	0.0b	0.9c	0.8f	0.3d	21.4
Chato	48	42.9b	0.0b	22.3a	23.5b	15.9b	59.4
Hombolo	46	4.0f	0.0b	0.0c	5.4ef	1.6d	0.0
Kizimbani	51	7.1d	0.0b	0.0c	2.3f	0.0d	38.1
Maruku	51	0.3g	0.0b	0.0c	12.1cd	3.8c	0.0
Naliendele	47	5.5de	2.0a	*	8.6de	4.8c	16.3
Suluti	43	0.2g	0.0b	0.0c	10.1cd	6.2c	0.0
Ukiriguru	48	11.4c	1.1ab	2.5c	16.4c	4.2c	56.3
**Mean/ total**	**435**	**17.3**	**0.6**	**5.2**	**12.3**	**6.7**	**30.5**

*No data, **N** = number of means, **Mean *Bemisia tabaci*** = *Bemisia tabaci* abundance (insects were counted from five fully expanded shoot tip leaves of the tallest shoot of each of ten plants selected randomly in a net plot. Averages of these counts were used to estimate the number of insects per plant [i.e. abundance] and to calculate the overall means per site, **CBSD 2MAP** = Percentage of cassava plants showing cassava brown streak disease leaf symptoms at two months after planting, **CBSD 12MAP** = Percentage of cassava plants showing cassava brown streak disease leaf symptoms at 12 months after planting, **Root incidence** = Percentage of cassava roots showing cassava brown streak disease root necrotic symptoms at harvest (12MAP), **Unusable root inc.** = Percentage of cassava roots showing cassava brown streak disease root necrotic symptoms that cannot be used for consumption or marketed, **CBSIs in roots** = Percentage of cassava roots infected by cassava brown streak ipomoviruses

**Table 4:** *Bemisia tabaci* abundance and cassava brown streak disease symptoms of selected cultivars planted at nine sites in Tanzania, November 2015-April 2017

Cultivar	N	*Mean *Bemisia tabaci* abundance	CBSD inc. 2MAP (%)	^#^CBSD inc. 12MAP (%)	*Root inc. (%)	*Unusable roots (%)	CBSIs in roots (%)
*Albert*	22	12.5 abcd	0.4	4.8 abc	13.5 bcd	9.9 cd	17.8
*Cho5_203*	27	14.1 bcd	0.8	14.7 a	28.6 a	23.0 a	43.8
*Eyope*	26	25.9 ab	0.8	2.7 abc	3.9 cde	1.7 efg	25.7
*F10_30R2*	25	16.9 abcd	0.0	0.0 c	9.8 bcde	3.3 cdefg	27.6
*Kalawe*	23	25.1 abc	2.4	15.7 ab	8.7 bcde	5.6 cdefg	24.4
*Kipusa*	27	27.2 ab	0.4	1.9 bc	15.1 bcd	6.4 cde	13.0
*Mkombozi*	25	20.4 abc	0.0	6.9 abc	14.4 bcd	6.4 cdef	31.0
*Mkumba*	26	9.4 d	0.8	0.0 c	7.2 bcde	0.6 fg	38.5
*Mkuranga1*	27	10.3 d	0.0	1.4 bc	3.2 de	0.9 fg	35.0
*Narocass1*	26	11.6 bcd	0.0	1.3 bc	4.8 cde	0.4 g	31.0
*Nase14*	26	20.7 abc	2.0	3.4 abc	14.2 bc	6.2 cde	30.7
*Nase18*	27	12.5 bcd	0.0	3.3 abc	14.9 b	8.0 cde	36.6
*Nase3*	25	16 bcd	0.0	4.5 abc	12.0 bcde	5.7 cdef	32.9
*Orera*	25	18.9 ab	0.4	1.3 bc	2.9 e	0.8 fg	14.6
*Sagonja*	27	28.3 a	1.1	12.5 abc	11.1 bcde	7.5 cde	41.7
*Sauti*	27	15.6 abc	0.4	11.0 abc	19.2 bc	14.5 bc	39.2
*Shibe*	25	16.5 abcd	0.4	3.3 abc	28.0 a	15.1 ab	35.9
**Mean/total 436**		**17.8**	**0.6**	**5.3**	**12.4**	**6.8**	**30.5**

*Values with the same letter are not significantly different; *P < 0.05*, ^#^Values with the same letter are not significantly different; *P = 0.05,***N**=number of means, **Mean *Bemisia tabaci* abundance** = *Bemisia tabaci* abundance estimated as the average number of insects per plant, **CBSD inc. 2MAP (%)** = Percentage of cassava plants showing cassava brown streak disease leaf symptoms at two months after planting, **CBSD inc. 12MAP (%)** = Percentage of cassava plants showing cassava brown streak disease leaf symptoms at 12 months after planting, **Root inc. (%)** = Percentage of cassava roots showing cassava brown streak disease root necrotic symptoms at harvest (12MAP), **Unusable roots (%)** = Percentage of cassava roots showing cassava brown streak disease root necrotic symptoms that cannot be used for consumption or marketed, **CBSIs in roots (%)** = Percentage of cassava roots infected by cassava brown streak ipomoviruses

CBSD leaf symptoms were observed at all but four sites: Hombolo, Kizimbani, Maruku and Suluti. These were the four sites with the lowest surrounding CBSD values. Significant differences in CBSD leaf incidence were observed across sites at 2MAP (F = 2.24, *P =* 0.03) and at 12MAP (F = 7.97, *P* < 0.0001; Table 3). The most affected sites were from the LZ where the highest incidences were recorded in Bunda followed by Chato – both at 2 and 12MAP. No significant differences were observed among cultivars at 2MAP, but significant differences were observed at 12MAP (F = 2.74, *P =* 0.003; Table 4). While no significant differences were observed between sites or cultivars for CBSD leaf symptom severity, all cultivars except *F10_30R2* expressed mild to severe symptoms (Table 5).

**Table 5:** Cassava brown streak disease leaf and root severities of selected cultivars planted at nine sites in Tanzania, November 2015-April 2017

Cultivar	N	fSev. 2MAP	fSev. 12MAP	^#^rSev.
*Albert*	27	2.00	2.95	2.70 abc
*CHO5_203*	27	2.50	2.70	3.10 a
*Eyope*	27	2.00	3.11	2.50 abcde
*F10_30R2*	27	*	*	2.30 bcde
*Kalawe*	27	2.67	2.88	2.60 abcd
*Kipusa*	27	2.00	2.00	2.50 abcde
*Mkombozi*	27	*	3.97	2.30 cde
*Mkumba*	27	2.50	*	2.10 de
*Mkuranga1*	27	*	3.67	2.10 de
*Narocass1*	27	*	3.67	2.10 e
*Nase14*	27	2.50	2.83	2.60 abcd
*Nase18*	27	*	2.33	2.40 bcde
*Nase3*	27	*	3.00	2.50 bcde
*Orera*	27	2.00	2.67	2.30 cde
*Sagonja*	27	2.33	3.99	2.60 abcd
*Sauti*	27	2.00	3.65	2.90 ab
*Shibe*	27	4.00	3.25	2.70 abc
**Overall Mean/Total**	459	**2.42**	**3.18**	**2.42**

**^#^**Values with the same letter are not significantly different; *P < 0.05*, *indicates no cassava brown streak disease symptoms were observed, **N** = number of entries, **fSev. 2MAP** = cassava brown streak disease (CBSD) leaf severity symptoms at two months after planting (MAP), **fSev. 12MAP** = CBSD leaf severity symptoms at 12MAP, **rSev.** = CBSD root severity symptoms recorded at harvest.

CBSD root symptoms were observed in all of the experimental sites and for all cultivars. Fig. 3 illustrates root necrosis symptoms from selected sites and cultivars. Overall root severities were mild (average 2.42). However, several cultivars analyzed separately at different sites had severity scores >3 (Table 5). CBSD root severity varied significantly between sites (F = 15.6, *P* < 0.0001). Highest root severity scores were recorded for susceptible cultivars at sites in north-western Tanzania: Bunda (3.49), Chato (3.17) and Ukiriguru (2.99) (Table 6). *Cho5_203* was the cultivar with the highest overall root severity (3.1) and *Mkuranga1* with the least (average 2.1, but similar to *Mkumba* and *Mkuranga1*) (F = 6.97, *P* < 0.0001; Table 5). Root incidence differed significantly between sites (F = 6.38, *P* < 0.0001) as well as among cultivars (F = 4.61, *P <* 0.0001). Similarly, unusable root incidence was significantly different among sites (F = 14.83, *P* < 0.0001; Table 3) as well as among cultivars (F = 8.02, *P* < 0.0001; Table 4). Bunda, which was the site with the highest surrounding inoculum pressure, had the highest unusable root incidence (28.3%) followed by Chato (15.9%) in north-western Tanzania. Strong positive correlations were demonstrated between CBSD leaf and root incidences (root incidence: R = 0.82, *P =* 0.007; unusable root incidence: R = 0.87, *P =* 0.003).

**Table 6:** Cassava brown streak disease leaf and root severities of **^$^**selected cultivars at nine sites in Tanzania, November 2015-April 2017

Site	Agro-ecological zone	N	fSev. 2MAP	fSev. 12MAP	^#^rSev.
Bunda	Lake zone	18	2.29	3.86	3.49 a
Chambezi	Coast zone	18	*	2.75	2.10 bcd
Chato	Lake zone	18	*	2.95	3.17 a
Hombolo	Central zone	18	*	*	2.03 cd
Kizimbani	Coast zone	18	*	*	2.04 d
Maruku	Lake zone	18	*	*	2.34 bcd
Naliendele	Southern	18	3.00	*	2.89 abc
Suluti	Southern	18	*	*	2.52 bcd
Ukiriguru	Lake zone	18	3.00	2.77	2.99 ab
**Overall Mean/Total**		**162**	**2.55**	**3.24**	**2.77**

**^#^**Values with the same letter are not significantly different; *P* < 0.05, *indicates no cassava brown streak disease symptoms were observed, **^$^**CBSD susceptible cultivars: *Albert*, *Cho5_203*, *Sagonja*, *Sauti* and *Shibe*, **N** = number of entries, **fSev. 2MAP** = cassava brown streak disease (CBSD) leaf severity symptoms at two months after planting (MAP), **fSev. 12MAP** = CBSD leaf severity symptoms at 12MAP, **rSev.** = CBSD root severity symptoms recorded at harvest.

**Fig. 2:**
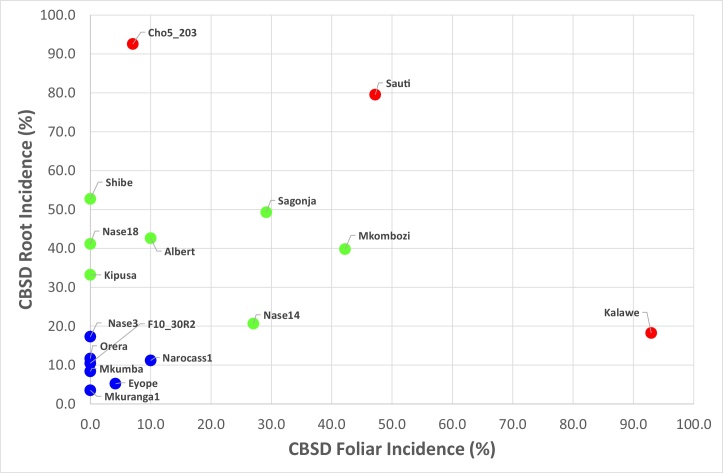
Relationship between foliar and root incidences of CBSD for cultivars evaluated at the high CBSD inoculum pressure location of Bunda, north-western Tanzania, 2015-2017. Cultivars with both mean values of incidence < 20% coloured blue, cultivars with one or both incidences > 20% but both less than 60% coloured green, cultivars with one or both incidences > 60% coloured red.

The relative patterns of CBSD symptom expression in leaf and roots can be compared for cultivars under the high inoculum pressure conditions experienced at Bunda (Fig. 2). Although the correlation between leaf and root incidence is clear, it is notable that some cultivars had high foliar incidence but low root incidence (e.g. *Kalawe*), whilst others had the inverse pattern (e.g. *Cho5_203*). This approach can be used to classify the cultivars for their response to CBSD. The seven top performers based on the criteria of lowest root and foliar incidence of CBSD were: *Mkumba*, *Eyope*, *Orera*, *Mkuranga1*, *Narocass1*, *F10_30R2* and *Nase 3*.

**Fig. 3:**
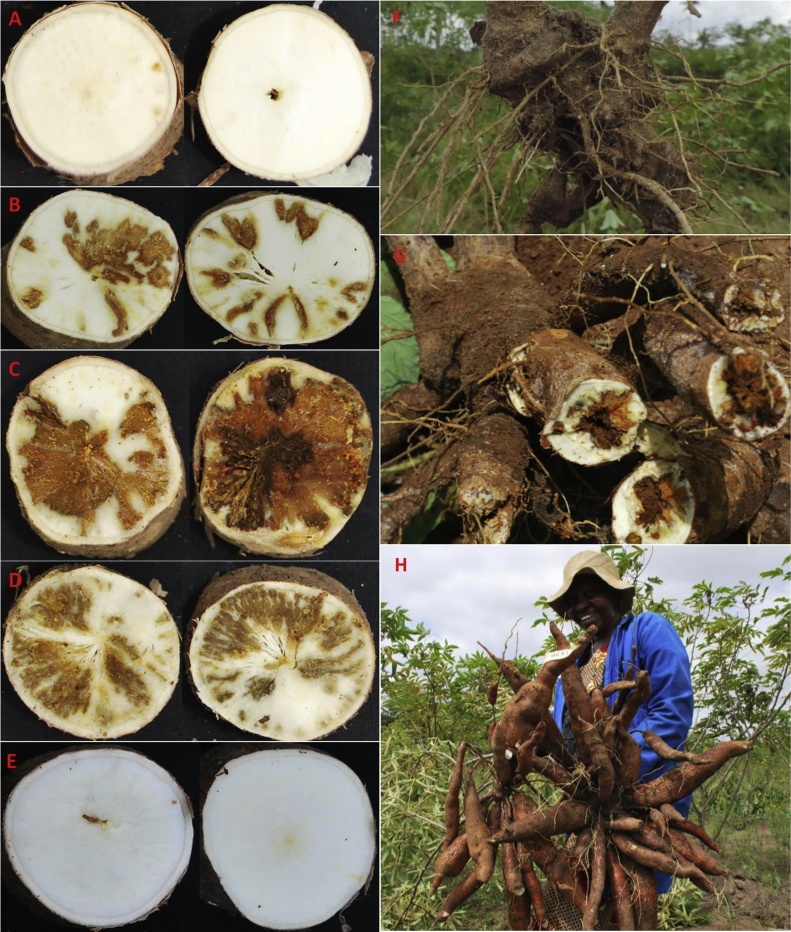
Cassava brown streak disease necrotic rot symptoms recorded after making cross-sectional cuts into the roots of selected cassava cultivars at Bunda site in north-western Tanzania. Panels A-E: *Mkuranga1*, *Albert*, *Cho5_203*, *Sagonja*, *Narocass1*; Panels F and G: cassava plants showing severely reduced (*Kalawe*, at Bunda) and damaged (*Cho5_203*, at Naliendele) roots, Panel H: Healthy roots of the cultivar *Narocass1* at Naliendele.

### 3.3 Real-time RT-PCR testing for CBSIs

Although CBSIs were detected in relatively equal proportions in fields surrounding the trial plots, CBSV was much more frequently detected in the trial plots themselves, since it accounted for >80% of all tested samples both for leaf and root testing. Overall, a relatively low proportion (<10.0%) of the leaf samples tested from trial plots gave positive tests. For root testing, infections were detected for all sites except Hombolo, Maruku and Suluti (Table 3). The greatest proportions of infected roots were observed in Bunda (74.7%) followed by Chato (59.4%). By contrast, few cultivars were affected in the coastal sites of Chambezi, Kizimbani and Naliendele. Using root sample testing data, all of the cultivars evaluated in this study were found to be infected by CBSIs (Table 4). Cultivar infection was most widespread at Bunda and Chato in north-western Tanzania, where all cultivars were infected.

### 3.4 Root yields

No evidence of cultivar by site interaction was demonstrated for root yield. Differences in fresh root yield among cultivars within sites were also not significant. There were, however, significant differences in fresh root yield (F = 10.49, *P* < 0.0001) among sites (Table 7) as well as amongst cultivars (F = 4.12, *P* < 0.0001) across sites (Table 8). The highest root yield was recorded at Naliendele (21.7 t/ha) while the lowest fresh root yield was recorded in Bunda (8.0 t/ha) (Table 7). Root dry matter content (DM) differed significantly across sites (F = 146.19, *P* < 0.0001). The greatest DM (35.0%) was recorded in Kizimbani while the lowest (20.1%) was in Hombolo while root harvest index (HI) was significantly higher in Suluti (0.63) compared to the lowest observed in Bunda (0.43; F = 4.64, *P =* 0.0003; Table 7). Significant differences were also observed for marketable yield. Kizimbani had 100.0% marketable yield whilst Bunda had the lowest (76.2%) (Table 7). Cultivars differed significantly in the amount of fresh root yield (Table 8, Table 9) where *Narocass1* (21.0 t/ha) had the highest whilst the lowest yield (10.7 t/ha) was recorded for *F10_30R2*. Similarly, significant differences (F = 3.63, *P* < 0.0002) were observed in marketable yield between cultivars (Table 8, Table 10) where *Narocass1* (20.9 t/ha) had the highest while *F10_30R2* (10.2 t/ha) had the lowest. However, whereas most of the cultivars had high percentages (>90.0%) of marketable yield, cultivars that were most affected by CBSD had lower percentages of marketable yield: *Cho5_203* (79.9%), *Shibe* (84.4%) and *Sauti* (85.4%) (Table 8).

**Table 7:** Fresh root yield, dry matter content and root harvest index of selected cassava cultivars evaluated at nine sites in Tanzania, 2015 – 2017

**Site**	**N**	*FRY, t/ha	*%DM	*Root HI	*%Marketable yield
Bunda	51	8.0 f	28.9 c	0.43 e	76.2 e
Chambezi	50	18.5 bc	29.8 c	0.52 bc	99.7 a
Chato	51	9.3 ef	29.5 c	0.43 e	84.1 d
Hombolo	47	11.9 de	20.1 e	0.46 de	98.4 a
Kizimbani	51	10.8 ef	35.0 a	0.51 bc	100.0 a
Maruku	51	16.6 c	34.5 ab	0.55 b	96.2 b
Naliendele	47	21.7 a	26.9 d	0.49 cd	95.2 b
Suluti	43	20.2 ab	33.5 b	0.63 a	93.8 c
Ukiriguru	48	14.1 d	26.9 d	0.49 cd	93.7 c
**Mean/Total**	**439**	**14.4**	**29.5**	**0.50**	**92.9**

*Values with the same letter are not significantly different; *P < 0.05*, **N** = number of plots, **FRY** = Fresh root yield measured, %**DM** = Percentage dry matter content, **HI** = Harvest index, %**Marketable yield** = Percentage of roots that are marketable.

**Table 8:** Fresh root yield, dry matter content and root harvest index of selected 17 cassava cultivars evaluated in Tanzania, 2015 – 2017

Cultivar	N	*FRY, t/ha	*%DM	*Root HI	*%Marketable yield
*Albert*	22	15.5 bc	31.8 a	0.51 bcde	90.1 e
*Cho5_203*	27	16.5 bc	28.5 abc	0.45 ef	76.9 g
*Eyope*	26	11.4 c	28.5 d	0.49 bcde	98.2 abcd
*F10_30R2*	25	10.7 c	31.3 ab	0.43 f	96 abcde
*Kalawe*	26	13.8 c	29.0 bcd	0.46 def	94.3 abcde
*Kipusa*	27	11.3 c	30.7 abc	0.47 cdef	93.6 cde
*Mkombozi*	25	15.1 bc	25.6 e	0.54 bc	93.5 bcde
*Mkumba*	26	13.5 c	31.9 a	0.50 bcdef	98.8 abc
*Mkuranga1*	27	12.8 c	29.9 abcd	0.47 cdef	99.1 a
*Narocass1*	26	21.0 a	28.4 cd	0.70 a	99.6 a
*Nase14*	26	11.8 c	28.0 d	0.53 bcd	92.8 e
*Nase18*	27	14.6 bc	29.7 abcd	0.53 bcd	91.8 e
*Nase3*	25	14.6 bc	30.9 abc	0.56 ab	94.1 bcde
*Orera*	25	11.7 c	30.0 abcd	0.42 f	99.2 ab
*Sagonja*	27	16.1 bc	29.7 abcd	0.47 cdef	92.4 de
*Sauti*	27	15.1 bc	29.0 bcd	0.48 cdef	85.4 ef
*Shibe*	25	19.7 ab	29.5 abcd	0.56 ab	84.4 fg
**Overall Mean/Total**	**439**	**14.4**	**29.5**	**0.50**	**92.9**

*Values with the same letter are not significantly different; *P < 0.05*, **N** = number of means, **FRY** = Fresh root yield measured, %**DM** = Dry matter content, **HI** = Harvest index, %**Marketable yield** = Percentage of roots that are marketable.

**Table 9:** Fresh root yield (t/ha) of cassava cultivars evaluated at nine sites in Tanzania, 2015-2017

Cultivar	Bunda	Chambezi	Chato	Hombolo	Kizimbani	Maruku	Naliendele	Suluti	Ukiriguru	Average*
*Albert*	11.1	11.2	9.1	8.0	14.7	17.7	23.0	36.8	14.4	15.5 bc
*Cho5_203*	4.3	20.1	4.0	11.4	17.9	25.4	28.8	23.0	13.7	16.5 bc
*Eyope*	8.2	12.5	7.2	9.4	6.9	12.4	19.8	14.8	11.6	11.4 c
*F10_30R2*	7.4	14.0	9.6	9.7	6.3	8.7	16.7	12.1	14.5	10.7 c
*Kalawe*	7.9	19.2	8.6	9.5	5.7	27.6	17.3	17.1	10.2	13.8 c
*Kipusa*	6.8	16.0	10.6	5.6	6.0	11.0	22.6	11.1	12.1	11.3 c
*Mkombozi*	4.7	25.1	7.9	16.5	11.7	19.3	20.6	18.5	14.8	15.1 bc
*Mkumba*	7.7	21.0	9.0	4.7	9.2	20.6	16.4	11.9	17.9	13.5 c
*Mkuranga1*	6.1	20.9	9.3	12.0	6.5	13.1	17.0	18.8	11.4	12.8 c
*Narocass1*	15.9	29.8	14.6	16.9	8.2	19.9	30.3	31.3	20.4	21.0 a
*Nase14*	6.8	13.1	8.8	12.1	7.8	7.9	20.5	16.6	14.1	11.8 c
*Nase18*	8.0	11.7	8.3	15.1	13.4	19.3	27.1	15.8	12.8	14.6 bc
*Nase3*	6.2	19.0	9.3	21.6	9.1	9.5	22.4	23.1	16.6	14.6 bc
*Orera*	12.7	13.2	9.7	11.4	9.7	13.2	13.5	12.6	10.3	11.7 c
*Sagonja*	5.5	23.2	12.4	10.4	10.3	18.7	19.4	30.2	15.1	16.1 bc
*Sauti*	3.7	19.3	14.0	9.8	17.0	9.7	23.8	25.5	12.9	15.1 bc
*Shibe*	12.9	23.7	5.4	19.6	23.4	27.7	28.1	21.7	15.7	19.7 ab
**Overall Mean**	**8.0**	**18.5**	**9.3**	**11.9**	**10.6**	**16.6**	**21.7**	**20.2**	**14.1**	**14.4**

* Values with the same letter are not significantly different; *P < 0.05*.

**Table 10:** Mean marketable fresh root yield (t/ha) of cassava cultivars evaluated at nine sites in Tanzania, November 2015-April 2017

Cultivar	Bunda	Chambezi	Chato	Hombolo	Kizimbani	Maruku	Naliendele	Suluti	Ukiriguru	Average*
*Albert*	7.0	10.9	7.8	8.0	14.7	17.7	18.2	33.8	13.9	14.0 bc
*Cho5_203*	0.4	19.8	1.7	11.4	17.8	25.1	19.6	22.3	10.6	14.3 bc
*Eyope*	7.9	12.5	7.1	9.1	6.9	11.9	19.7	14.5	11.2	11.2 c
*F10_30R2*	7.2	14.0	8.1	9.6	6.3	8.5	16.7	11.4	13.0	10.2 c
*Kalawe*	7.1	19.2	6.7	9.5	5.7	22.1	16.4	17.1	10.0	12.7 bc
*Kipusa*	6.3	16.0	7.7	5.6	6.0	10.6	22.5	9.8	11.8	10.7 c
*Mkombozi*	2.7	25.1	7.1	16.5	11.7	19.1	20.6	16.1	14.3	14.5 bc
*Mkumba*	7.7	21.0	8.9	4.7	9.2	20.0	16.2	11.9	17.1	13.3 bc
*Mkuranga1*	6.1	20.9	8.7	11.9	6.5	12.8	17.0	18.8	11.4	12.7 bc
*Narocass1*	15.6	29.8	14.5	16.9	8.2	19.9	30.3	31.3	20.3	20.9 a
*Nase14*	6.1	13.1	7.8	11.0	7.8	7.6	18.8	16.0	12.3	11.0 c
*Nase18*	4.3	11.7	6.7	15.1	13.4	19.1	26.2	14.9	12.0	13.7 bc
*Nase3*	5.7	19.0	8.1	20.5	9.1	9.4	22.4	20.8	14.3	13.8 bc
*Orera*	12.1	13.2	9.5	11.4	9.7	13.2	13.4	12.6	10.3	11.6 c
*Sagonja*	3.9	23.2	10.9	10.4	10.3	18.7	18.1	29.2	14.3	15.4 bc
*Sauti*	0.8	19.3	7.0	9.7	17.0	9.7	23.8	20.1	12.4	13.3 bc
*Shibe*	8.1	23.7	4.3	17.4	23.3	19.6	27.2	14.4	13.9	16.9 b
**Overall Mean**	**6.4**	**18.5**	**7.8**	**11.7**	**10.8**	**15.6**	**20.5**	**18.8**	**13.2**	**13.5**

* Values with the same letter are not significantly different; *P < 0.05*. Figures represent fresh root yield measured in t/ha.

There was no relation between dry matter and CBSD. P values for correlations between harvest index and CBSD incidences (foliar, root, unusable root and CBSIs in roots) were all greater than 0.9. By contrast, correlations between harvest index and foliar CBSD incidence (coefficient = -0.632; *P =* 0.068) and CBSIs in roots (coeff. = -0.655; *P =* 0.056) were marginally non-significant. Percentage of marketable roots was negatively correlated with CBSD foliar incidence (coeff. = -0.863; *P =* 0.0027), root incidence (coeff. = -0.965; *P =* 0.000025) and CBSIs in roots (coeff. = -0.730; *P =* 0.025). Fresh root yield was negatively correlated with CBSIs in roots (coeff. = -0.687; *P =* 0.041), whilst marketable root yield was negatively correlated with both foliar incidence (coeff. = -0.672; *P =* 0.047) and CBSIs in roots (coeff. = 0.679; *P =* 0.045).

## 4 Discussion

A multi-location evaluation of elite cassava cultivars was conducted in Tanzania between November 2015 and April 2017 during which 17 cultivars (including a CBSD-susceptible landrace [*Albert*]) were evaluated at each of nine sites. Results of this study highlighted the importance of surrounding inoculum and the abundance of whitefly vectors in the spread of CBSD into experimental fields. Disease spread differed widely depending on relative cultivar resistance/susceptibility to CBSIs and the characteristics of the site where they were planted. Although the most resistant cultivars yielded significantly more than the most susceptible cultivars at the highest disease pressure locations, susceptible cultivars gave some of the highest yields where disease pressure was low. These results thus highlight the importance of applying a balanced strategy to CBSD management that seeks to enhance resistance whilst also making use of yield and quality traits present in local landraces and applying phytosanitary control including the use of disease-free planting material and picking optimal planting dates. The study reported here was part of a regional evaluation trial of elite cassava cultivars across diverse environments in five countries in East and Southern Africa (IITA, 2012; Tumwegamire et al. 2018) and made use of several of the most promising putative CBSD-resistant cultivars available from each of those five countries. Nine study sites were carefully selected to cover the major cassava-producing agro-ecological zones in Tanzania, which were anticipated to have contrasting CBSD inoculum conditions.

1. Differences in CBSD infection are driven by whitefly abundance and CBSD inoculum pressure

CBSD inoculum pressure was highest in the LZ in north-western Tanzania. Although similar conditions were observed at Chambezi in the CZ, inoculum pressure in central, coastal and southern Tanzania was generally lower than that in the LZ. Sites with highest whitefly abundances were also recorded in the north-western region. Virus transmission and disease spread to new sites are determined by inoculum source (Legg et al. 1997), proximity and vector abundance (Legg et al. 2011, 2017). Therefore, the high CBSD pressure recorded for some of the sites in this study meant that higher virus transmission rates were experienced at those sites. Studies have also shown that efficient transmission of pathogens or disease spread are tightly linked to prevailing environment and/or growing season (Shirima et al. 2019) and crop age (Fishpool et al. 1995; Legg (1995)), with each having important effects on whitefly vector abundance. Data in this study demonstrated very clearly the importance of the twin factors of whitefly abundance and surrounding CBSD inoculum pressure in driving CBSD infection of initially CBSD-free trial plantings. The two sites where both CBSD inoculum pressure and vector abundance were high had much greater levels of CBSD infection in trial plots than all other sites. By contrast, there were much lower levels of CBSD infection at sites that had high surrounding CBSD but few vectors (Chambezi – CZ) or abundant vectors but little surrounding CBSD (Maruku – LZ). These results highlight the importance of assessing inoculum pressure where cultivars are to be evaluated for their resistance to CBSD.

Some of the sites were significantly less affected by CBSD than might have been anticipated. Chambezi, near Bagamoyo in the CZ, is used by breeders as a high CBSD pressure location. It was notable that its surrounding CBSD value was one of the highest recorded. However, *B. tabaci* abundance was extremely low. Kizimbani, on the island of Zanzibar, similarly had low whitefly abundances. The likely reason for the low whitefly populations observed at both sites is the planting of the trial during the main rainy season. Data from trials planted in this agro-ecological zone in both the main rains (March-June) and the short rains (October-December) demonstrate that whitefly abundance and concomitant CBSD spread are much greater in the short than the long rains (Shirima et al. 2019).

2. Patterns of CBSD resistance differ in cassava roots and shoots

Cultivars responded differently to CBSIs in expressing shoot symptoms across sites, but there was clear evidence demonstrating that some of the cultivars were less readily infected by CBSIs than others. Few cultivars remained asymptomatic in shoot symptom components across all sites throughout the study period while some had mild and others had severe shoot symptoms.

Root symptoms were observed in all sites and cultivars. Differences in patterns of root and shoot symptom expression between cultivars highlight an important question concerning the manner in which mechanisms of resistance function. The generally higher levels of root incidence compared to shoot incidence observed in this experiment contrasts with a previous study (Ndyetabula et al. 2016) where CBSD shoot incidences were higher. However, the result of the current study is comparable to that of previous research in coastal Tanzania (Shirima 2019) where root incidence was higher than shoot incidence. It is worth noting that for Ndyetabula et al. (2016), assessments were conducted during a survey when 9-10-month-old plants were sampled. A well-known feature of CBSD is that root necrosis symptoms become increasingly severe as the plant matures towards and beyond normal harvest age (12 months) (Nichols, 1950). It appears likely, therefore, that root incidences in the 2016 study were underestimated as a result of the premature harvesting for root assessment.

Plotting foliar against root incidences of CBSD for cultivars evaluated at the high CBSD inoculum pressure location of Bunda illustrated the generally strong correlation between these two measures of CBSD, although two of the most susceptible cultivars had divergent responses – one with high foliar incidence but low root incidence (*Kalawe*) and the other with low foliar incidence and high root incidence (*Cho5_203*). Variability in patterns of symptom expression between cultivars is a phenomenon that was noted from some of the earliest studies (Nichols, 1950), and has been confirmed in quantitative terms more recently (Ndyetabula et al. 2016). This latter study which surveyed farmer-grown cultivars in Tanzania noted that whilst cultivar *Lyongo* had moderate foliar symptom incidences yet > 80% incidence of root symptoms, cultivar *Kiroba* had high incidences of both leaf and stem symptoms but < 10% incidence of root symptoms. In the same study, district-level incidences of foliar, root and unusable root symptoms were used to define ‘resistance’ and ‘tolerance’ variables, which when plotted on y and x axes enabled cultivars to be categorised into four groups, with Category I having the best combination of ‘resistance’ and ‘tolerance’ and Category IV the worst. In the current study, an alternative approach to categorising CBSD response was used and three categories were defined based on the combination of foliar and root incidence values. Perhaps fortuitously, the three categories were each clearly delimited, and seven of the 17 cultivars assessed fell within the top-performing category, with both foliar and root incidences of less than 20%. One cultivar in the current study (*Mkombozi*) was also represented in that of Ndyetabula et al. (2016). Although this was amongst the top-performing Category I cultivars in the 2016 study, it was one of the poorest performers of the middle category in the current study. This demonstrates the much higher overall level of resistance to CBSD of the cultivars tested here when compared with the larger set of primarily farmer-grown local landraces of the 2016 study. As such, it is an important confirmation of the value of the conventional CBSD resistance breeding work being undertaken in East and Southern Africa.

3. Cassava brown streak ipomoviruses and CBSD aetiology

Most of the CBSD root necrosis symptoms reported in this study were linked to CBSIs (confirmed by specific PCR assays). However, no CBSIs were detected in either leaf or root samples at three sites: Hombolo, Suluti and Maruku, where significant incidences of CBSD root symptoms were observed. Furthermore, none of the cultivars expressed foliar symptoms at these three sites. These results highlight the need for further scrutiny to determine whether there are other pathogens or physiological factors causing CBSD-like symptoms in cassava roots. CBSIs were more frequently detected in roots than in leaves, as observed previously in Uganda (Ogwok et al. 2015). CBSV was the major species detected in sites in north-western Tanzania while UCBSV was most frequent in samples collected in the CZ sites, albeit at low incidences (<10%). Mixed infections were rare (detected in only two sites). CBSV and UCBSV were detected almost equally at the outset in surrounding fields but results of this study suggest that CBSV is either more efficiently transmitted by the whitefly vector, *B. tabaci*, since it was the virus that was most frequently detected in the experimental plots, or cultivars have a generally higher level of resistance to UCBSV. Controlled studies of the transmission of CBSI species by *B. tabaci* did show a slightly higher efficiency in transmission of CBSV compared to UCBSV (Maruthi et al. 2017), although the differences were not significant. These experiments did involve relatively small numbers of replicates, however, and it may be that had there been greater replication a significant result would have been obtained. In the current study, the high number (>90%) of infected cultivars in north-western Tanzania where CBSV was the most frequent virus probably confirms reports from Uganda and from laboratory studies that the virus is more virulent than its sister species, UCBSV (Kaweesi et al. 2014; Mohammed et al. 2012; Winter et al. 2010). We cannot confirm this for the current study, however, since sites in the CZ were planted during the long rainy season, which has been characterized as a low CBSD pressure season (Shirima et al. 2019) making comparisons difficult.

4. The impacts of CBSD are now greater in the LZ in north-western Tanzania than the CZ

In the current study, sites with the highest shoot incidences also had the greatest number of cultivars affected by CBSD (Bunda and Chato in north-western Tanzania). Whereas CBSD spread in this part of Tanzania is relatively recent compared to the CZ (Legg et al. 2011), results of this study suggest that trends in disease epidemiology are changing, and that CBSD incidences are increasing in north-western Tanzania in contrast to previous reports which suggested that CBSD was more important in the CZ (Jeremiah et al. 2015; Legg and Raya 1998; Ndyetabula et al. 2016). For most of its known history, CBSD has been confined to coastal East Africa and the shores of Lake Malawi (Nichols 1950; Hillocks and Jennings (2003)). Since 2004, however, CBSD has been spreading through the Great Lakes region (Alicai et al. 2007). From this first report from Uganda in 2004, subsequent spread has been reported into western Kenya, Tanzania, Rwanda, Burundi and eastern Democratic Republic of Congo (Tomlinson et al., 2018). The important change in the regional balance of the importance of CBSD within Tanzania highlights the expanding impact of the CBSD pandemic within parts of Africa that were previously unaffected. The pandemic of CBSD, and severe CMD before it, have been driven by greatly elevated populations of the whitefly vector, *B. tabaci* (Legg et al., 2011; 2014).

5. There were no differences in varietal performance across contrasting agro-ecological environments

Results from this study showed that although there were significant differences in fresh root yield for both site and cultivar, the differences between sites were much stronger and there was no significant ‘genotype by environment (GxE)’ interaction between the two factors. GxE interactions are widely reported to influence a variety of traits of cassava genotypes across contrasting agro-ecological environments (Esuma et al. 2016; Fotso et al. 2018), with the consequence that cultivars are often recommended for specific regions within countries. There are exceptions to this general theme within the published literature on cassava, however, and some studies have demonstrated the absence of GxE interactions for traits such as yield (Tumuhimbise et al. 2014) and starch quality (Karlström et al. 2019). The lack of a significant GxE interaction with respect to yield in our study is likely to be a consequence of multiple factors being associated with environment differences. The southern and eastern shores of Lake Victoria (Chato and Bunda sites) have low rainfall and poor soil fertility which are likely causes of the generally low yields for all cultivars at these sites. These also happened to be the sites with the greatest incidences of CBSD. Differences in performance resulting from the relative resistance/susceptibility of cultivars to CBSD may therefore have been masked by the combined effects of low rainfall and poor soil fertility. The strength of the contrast in site to site performance identified from this study emphasizes the importance of addressing soil moisture stress and fertility in parts of Tanzania where these have a large impact on cassava yields, such as in the LZ region of north-western Tanzania. On-going initiatives are designing site-specific fertilizer recommendations for these parts of the country, and a smartphone app has been developed (Akilimo-IITA, 2019) that allows extension officers, farmers or any other interested party to access agronomic advice of this type online.

6. Susceptible cultivars perform well in the absence of CBSD pressure and have important quality attributes

Three of the cultivars most affected by CBSD were *Cho5_203*, *Sagonja* and *Sauti*. These had the lowest mean yields of marketable fresh roots at the site most affected by CBSD (Bunda), yet all three had above average yields at one of the least affected sites – Suluti. *Cho5_203* was the most extreme example, as it had some of the best marketable fresh root yields at Maruku (25.1 t/ha) and Suluti (22.3 t/ha), yet had the lowest marketable yields of all 17 cultivars at both Bunda (0.4 t/ha) and Chato (1.7 t/ha). Similarly, *Albert*, which was also heavily affected by CBSD at Bunda and Chato, had the highest marketable fresh root yield of any cultivar at any site (33.8 t/ha at Suluti). A potential weakness of breeding programmes can be that cultivars that show susceptibility to target diseases at any site at any stage of the breeding pipeline are discarded. A second is that valuable traits of local landraces may be overlooked, as these genotypes may never be evaluated, or if they are, they are likely to be discarded in the early single-site stages of the breeding programme where that site is often chosen for its high disease pressure conditions. Another study from Tanzania, which further emphasizes this point, involved the evaluation of 64 local landraces at Naliendele (southern coastal region), eight of which gave higher marketable fresh root yields than the check improved cultivar – *Kiroba* (Masinde et al. 2018). *Kiroba* is currently one of the improved cultivars that is being heavily promoted in coastal Tanzania by research, extension and the private sector. A further significant finding from this study was that three local landraces were identified as the most resistant (Chimaje, Mfaransa and Supa B), with all shown to be significantly more resistant to CBSD than the improved cultivar check – *Kiroba*. The findings of this study, as well as our own, stress the value of exploiting local landraces within breeding programmes as sources of genes for disease resistance and high yield potential. Furthermore, organoleptic or other properties of local landraces are often cited by farmers as reasons for them being preferred over improved disease-resistant cultivars, even where the landraces yield less (Nakabonge et al. 2018).

7. Strategic considerations for deploying host plant resistance and other methods for the control of cassava brown streak disease

A balanced strategy for the most effective deployment of cassava cultivars to manage CBSD should be cognizant of the diversity of varietal responses to the disease under the widely contrasting agro-ecological and disease pressure conditions of a country such as Tanzania. This should include the promotion of disease-resistant released cultivars in regions most affected by CBSD coupled with the encouragement of phytosanitary control practices for existing cultivars in low disease pressure regions or in seasons during which there is reduced spread of CBSD. One of the key findings of a recent study at Chambezi in coastal Tanzania was that there was a high level of CBSD infection in cassava planted during the short rains (October-December), yet very little infection during the long rains (March-June) where vector abundance was low (Shirima et al. 2019). Important phytosanitary practices for CBSD management in susceptible cultivars can therefore include the selection of CBSD-free stems for replanting for low disease pressure regions and combining this tactic with planting during the long rains in areas with higher disease pressure. In the longer term, cultivar development teams should make use of all available germplasm sources in order to develop high yielding, disease-resistant cultivars with specific end-user quality traits, such as high starch content, amylose-free (waxy) starch, earliness, below-ground storability and resistance to post-harvest deterioration. Biotechnological approaches are already well advanced for cassava improvement and CBSD resistance has been one of the main targets for transgenic strategies in cassava. Success has been achieved in transforming cassava for resistance to the CBSIs (Ogwok et al. 2012) and cassava genotypes developed in this way have been shown to provide effective control when evaluated using confined field trials in East Africa (Wagaba et al. 2017). Although the genotypes used have either been model cultivars or other improved cultivars with existing resistance to CMD, there would also be value in using transgenic approaches to introduce CBSD resistance to susceptible landraces that have specific desirable end user quality traits. Although the technical capabilities are already in place in several African labs to do this, progress is currently constrained by regulatory concerns in many countries about genetic modification. Gene editing may offer a way to overcome this impasse, and the first proof-of-concept results have already been published describing the effectiveness of CRISPR/Cas9-mediated gene editing in reducing the severity of CBSD in infected plants of the model cultivar TMS 60444 (Gomez et al. 2018). Future developments in the application of these approaches are expected to deliver increased levels of CBSD resistance, and with anticipated improvements in the regulatory environment, there is likely to be strong potential for the production of new cultivars combining disease resistance with high yield and preferred quality traits. This study, which reports the first multi-location evaluation of elite cassava cultivars in Tanzania, offers a strategic benchmark for evaluating cassava performance in the future.

## Funding

This work was supported by the Bill & Melinda Gates Foundation, Seattle, WA [grant number OPP1022738].

## CRediT authorship contribution statement

**Rudolph R. Shirima:** Conceptualisation, Methodology, Investigation, Writing - review & editing. **James P. Legg:** Conceptualisation, Writing - review & editing, Supervision. **Daniel G. Maeda:** Conceptualisation, Writing - review & editing, Supervision. **Silver Tumwegamire:** Conceptualisation, Methodology, Writing - review & editing. **Geoffrey Mkamilo:** Conceptualisation, Methodology. **Kiddo Mtunda:** Methodology, Investigation, Writing - review & editing. **Heneriko Kulembeka:** Methodology, Investigation, Writing - review & editing. **Innocent Ndyetabula:** Investigation, Writing - review & editing. **Bernadetha P. Kimata:** Investigation, Writing - review & editing. **Dwasi Gambo Matondo:** Investigation, Writing - review & editing. **Gloria Ceasar:** Investigation, Writing - review & editing. **Edda Mushi:** Investigation, Writing - review & editing. **Karoline Sichalwe:** Investigation, Writing - review & editing. **Edward Kanju:** Conceptualisation, Methodology, Writing - review & editing.

## Declaration of Competing Interest

The authors declare no conflict of interest.
